# SAD-Based Stereo Vision Machine on a System-on-Programmable-Chip (SoPC)

**DOI:** 10.3390/s130303014

**Published:** 2013-03-04

**Authors:** Xiang Zhang, Zhangwei Chen

**Affiliations:** 1 State Key Laboratory of Fluid Power Transmission and Control, Zhejiang University, Hangzhou 310027, China; E-Mail: chenzw@zju.edu.cn; 2 School of Computer, Hangzhou Dianzi University, Hangzhou 310018, China

**Keywords:** stereo vision, system-on-programmable-chip, FPGA, disparity, SAD

## Abstract

This paper, proposes a novel solution for a stereo vision machine based on the System-on-Programmable-Chip (SoPC) architecture. The SOPC technology provides great convenience for accessing many hardware devices such as DDRII, SSRAM, Flash, *etc.*, by IP reuse. The system hardware is implemented in a single FPGA chip involving a 32-bit Nios II microprocessor, which is a configurable soft IP core in charge of managing the image buffer and users' configuration data. The Sum of Absolute Differences (SAD) algorithm is used for dense disparity map computation. The circuits of the algorithmic module are modeled by the Matlab-based DSP Builder. With a set of configuration interfaces, the machine can process many different sizes of stereo pair images. The maximum image size is up to 512 K pixels. This machine is designed to focus on real time stereo vision applications. The stereo vision machine offers good performance and high efficiency in real time. Considering a hardware FPGA clock of 90 MHz, 23 frames of 640 × 480 disparity maps can be obtained in one second with 5 × 5 matching window and maximum 64 disparity pixels.

## 1. Introduction

The major task of a stereo vision system is to reconstruct the 3D representation of the scene from the 2D images captured by those cameras which are fixed with their optical axes parallel and separated by a certain distance. The 3D information can be applied to complex tasks such as robot navigation systems, obstacle and lane detection, *etc.* [1,2].

Stereo matching algorithms have played an important role in stereo vision. They can be classified into either local or global methods of correspondence. Local methods match one window region centered at a pixel of interest in one image with a similar window region in the other image by searching along epipolar lines. The disparity is obtained by calculating the distance between two candidate window regions containing the most similarity. The performance of local stereo matching algorithms depends to a large extent on what similarity metric is selected. Typical similarity metrics are cross-correlation (CC), the sum of absolute differences (SAD), the sum of squared differences (SSD), the census transformation (CENS), *etc.* SSD and SAD find correspondences by minimizing the sum of squared or that of absolute differences in WxW windows.

As it is well known, the stereo matching algorithm is computationally and data intensive because it has to perform an identical operation on a large amount of pixels. Consequently, a special hardware system is most often required.

There are various examples of stereo vision algorithms implemented on FPGA in the literature. The circuit [3] is a stereovision system based on a Xilinx Virtex II using the SAD algorithm. The system can process images with a size of 270 × 270 at a frame rate of 30 fps. Paper [4] presents a FPGA-based stereo matching system that operates on 512 × 512 stereo images with a maximum disparity of 255 and achieves a frame rate of 25.6 fps running under a frequency of 286 MHz. In [5], a development system based on four Xilinx XCV2000E chips is used to implement a dense, phase correlation-based stereo system that runs at a frame rate of 30 fps for 256 × 360 pixels stereo pairs. Gardel *et al.* introduce in [6] their design, which can obtain 30,000 depth points from images of 2 Mpix at a frame rate of 50 frames per second under a 100 MHz working frequency. A real-time fuzzy hardware module based on a color SAD window-based technique is proposed in [7]. This module can theoretically provide accurate disparity map computation at a rate of nearly 440 frames per second without considering the memory delay and other factors of time consumption, thus giving a stereo image pair with a disparity range of 80 pixels and 640 × 480 pixels resolution. The design in [8] is a 7 × 7 binary adaptive SAD based real-time stereo vision architecture with a depth range of 80, which is implemented on the Altera Cyclone II EP2C70 FPGA chip based on 800 × 600 color images and operates in real-time at a frame of 56 Hz. The architecture captures the 90 Megapixel/sec 12 bit signals of two cameras in real-time and does not require memories external to the FPGA.

This paper proposes a new architecture that can solve the matching problem on variant image resolution of 256 × 256 to 695 × 555 pixels by using the SAD stereo matching algorithm. The hardware is based on SoPC technology and all circuits are implemented on a single Cyclone II FPGA chip. The Nios II processors, a configurable soft IP core, is added in this system to manage the buffer addresses of stereo images in SSRAM and to transfer the configuration data of users to other hardware modules through the Avalon bus interface. The disparity computation unit, modeled by the Matlab-based DSP Builder, is in charge of computing the SAD value of 5 × 5 pixel windows and extracting the disparity from the 64 candidates of SAD. The stereo matching controller, designed in the Verilog-HDL, is in charge of the update of the line buffer data in the on-chip dual-port RAM (DPRAM) and the write back disparities to the off-chip DDRII SDRAM. The whole system can produce 640 × 480 dense disparity maps at a frame rate of 23 fps under a 90 MHz working clock frequency.

## 2. DSP Builder Design Flow

**Figure 1.**
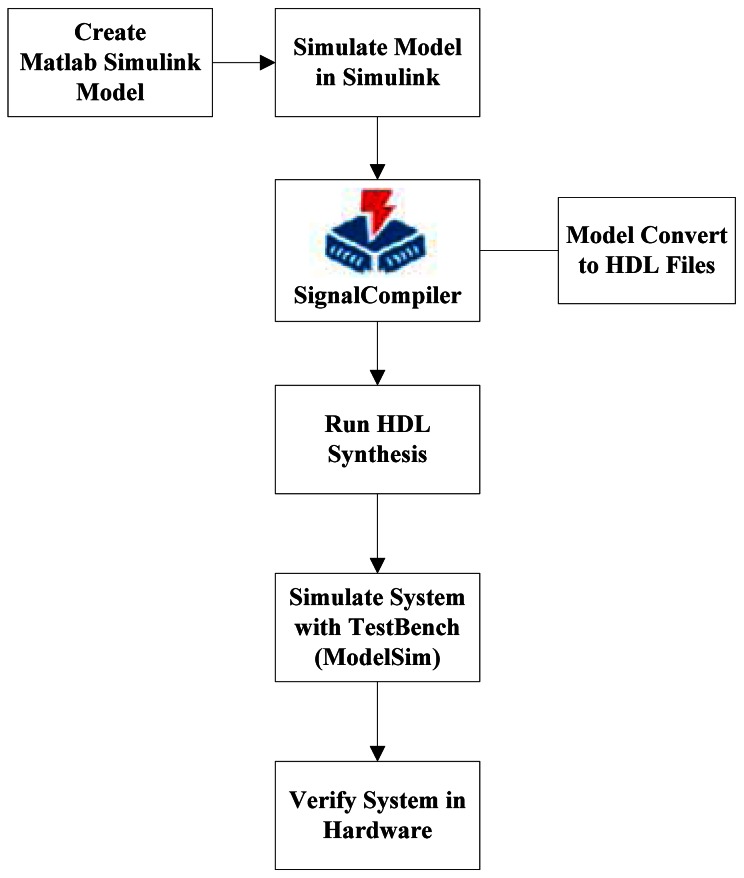
DSP Builder System-Level Design Flow.

DSP Builder integrates the algorithm development, simulation, and verification capabilities of MathWorks MATLAB and Simulink system-level design tools with the Altera Quartus II software and third-party synthesis and simulation tools. The DSP Builder works with the Simulink environment. The designer can combine Simulink blocks with the DSP Builder blocks to verify system level specifications and perform simulation. Figure 1 shows the DSP Builder system-level design flow.

The modules of our design mentioned in Section 4 are all modeled and simulated by Matlab/ Simulink. Researchers interested in those models can e-mail the author to ask for the mdl files. Usually, we model algorithm modules in Simulink, not the whole system. After automatic HDL generation, we can easily add the algorithm modules to the top-level design file of our system. We just need to add a *.qip file (Quartus II IP file) in the Quartus II project and instantiate an instance of the algorithm module in the top-level HDL file.

## 3. SoPC Architecture for SAD-Based Stereo Vision Machine

**Figure 2.**
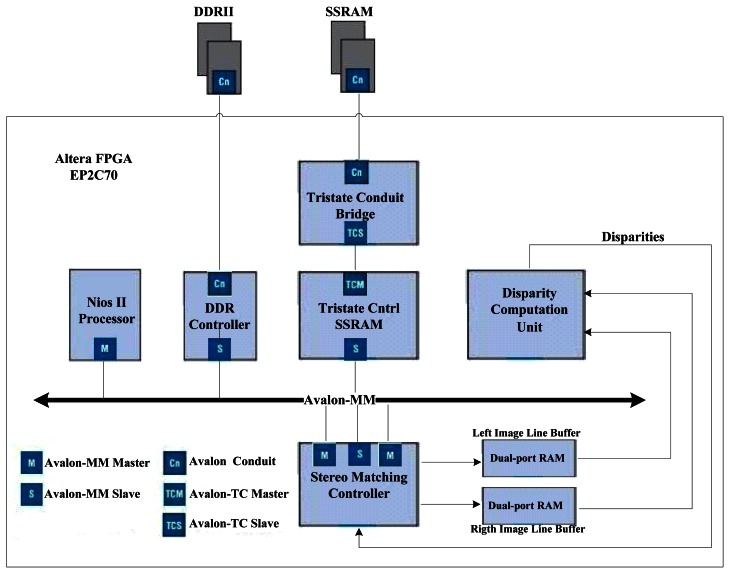
Proposed SoPC Architecture.

The system proposed herein is divided into the main modules as shown in Figure 2:

(a) Nios II processor system: It consists of a 32-bit Nios II processor core, a set of on-chip peripherals, on-chip memory and interfaces to off-chip memory.
(b) Disparity Computation Unit (DCU): This unit uses SAD as the similarity metric. After receiving the data of the stereo images, it calculates 64 SAD values and the output disparity within 6 clock-periods.
(c) Stereo Matching Controller (SMC): This unit involves two DMA engines. The first one reads data of the stereo images form SSRAM and writes them to two DPRAMs on the FPGA chip used as line buffers for the DCU. The second one reads disparities produced by the DCU and writes to the disparity table in the off-chip DDRII SDRAM. Two state machines are designed for the management of line buffers and the stereo matching processes.

The different modules of the system are interconnected with the Altera Avalon Memory-Mapped (Avalon-MM) interface applied for the read and write interfaces on master and slave components in a memory-mapped system [9]. The stereo images are stored in the off-chip SSRAM memory because it can offer a shorter read cycle than the DDRII SDRAM. The information about start address and resolution of images is passed to special function registers of the DCU through the Avalon interface by the C code executing on the Nios II processor. The SMC starts to initialize the line buffer of Left/Right images after the start bit of the system is set by the processor. Pixel data are sent from the line buffer to the DCU continuously till the whole dense disparity map is established.

## 4. Hardware Implementation of the Disparity Computation Unit

The SAD algorithm has the advantage of computational efficiency. The SAD equation used for 5 × 5 windows with a maximum disparity of 64 can be seen below:

(1) $$\text{SAD}(i,j,\text{disp})=\sum_{h=-2}^{2}\sum_{k=-2}^{2}|P_{R}(i+h,j+k)-P_{L}(i+h,j+k+\text{disp})|$$

where disp is the disparity value ranging from 0 to 63, *P_R_* (*i*, *j*) serves as the reference pixel in the right image and *P_L_* (*i*, *j*+*disp*) as the currently analyzed candidate pixel in the left image.

**Figure 3.**
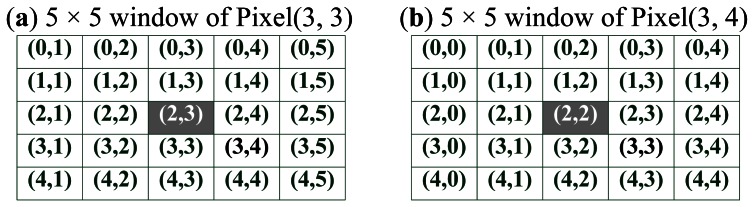
The Data Difference between two 5 × 5 windows of the adjacent pixels.

The reference 5 × 5 window centered at *P_r_*(*i*, *j*) is compared to 64 possible candidate windows to calculate 64 SAD values. There are 25 bytes of data for the right image and 340 bytes of data for the left image involved in the operation for the calculation of disparity(*i*, *j*), where disparity(*i*, *j*) means the disparity value of the pixel(*i*, *j*). It can be easily observed that different disparity calculations have many operations in common. For example, as show in Figure 3, the data involved with calculations of disparity(3,3) and disparity(3,4) differ from each other for only 10 bytes of data (5 bytes data of both the right and the left). We use two shift-taps to temporarily store the data used in calculating the disparity. The shift-tap for the right image has 25 taps and the other has 340 taps for the left. We propose two principles for feeding data to two shift-taps accordingly:

(a) The system transfers 25 bytes to the right shift-tap and 340 bytes to the left for the first pixel in every line.
(b) The system transfers 5 bytes of new data to the right and left shift-taps separately for the rest of the pixels in every line.

**Figure 4.**
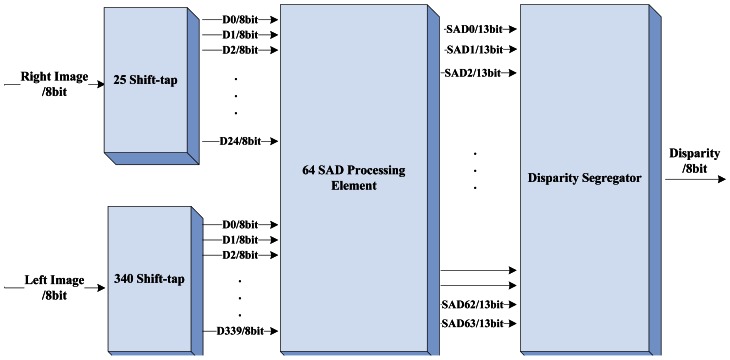
Block Diagram of the Disparity Computation Unit.

**Figure 5.**
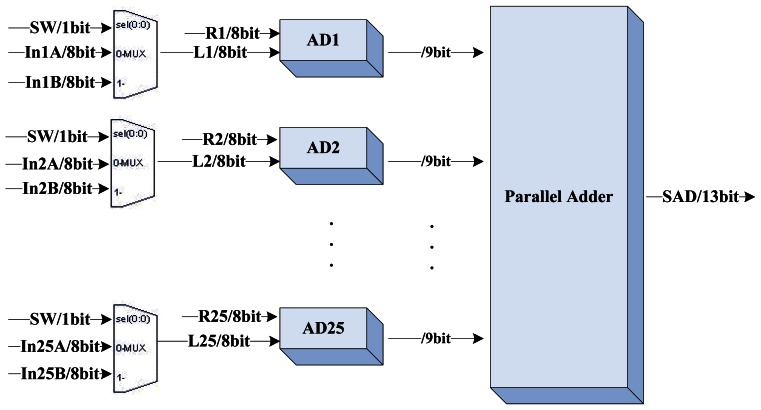
Layout Plan of the Parallel SAD Calculator.

**Figure 6.**
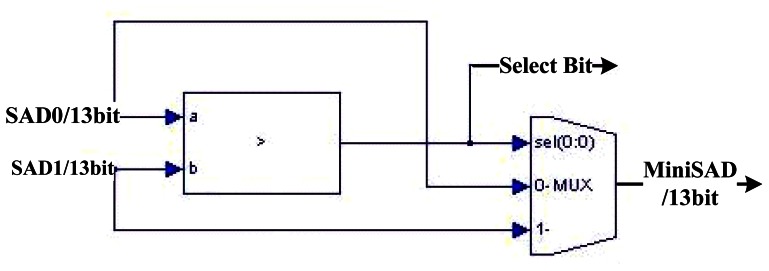
Basic Comparison Element of the DS Module.

**Figure 7.**
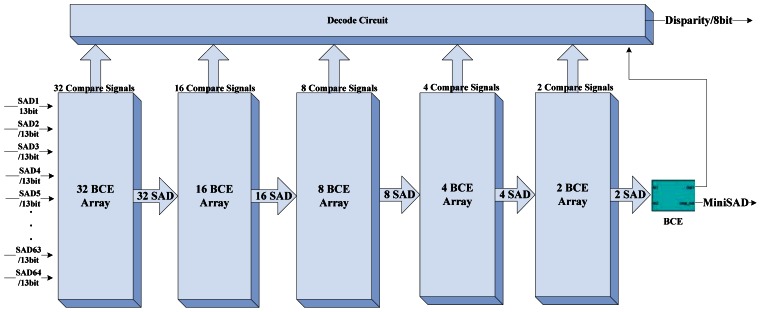
Diagram of the Disparity Segregator.

The block diagram of the DCU is shown in Figure 4. The two shift-taps receive image data from the buffer management unit in serial and feed to the 64 SAD processing element (SAD-PE) in parallel. The computation of the 5 × 5 SAD needs a 25 input parallel adder which costs too much logical elements in the FPGA, therefore we only arrange 32 parallel SAD calculators in the SAD-PE. Figure 5 illustrates the layout plan of the parallel SAD calculator. The SAD-PE can calculate 32 SAD values within 5 clock cycles (25-input parallel adder has four pipeline stages inside). With an input switch signal, it can finish 64 SAD computations in six clock cycles. Four additional clock cycles should be added for latching the 64 output SAD values separated. The Disparity Segregator (DS) module calculates the minimum SAD value using parallel comparators from 64 SAD values and outputs the index number as the disparity. Figures 6 and 7 are the detailed implementation of the DS module. All actions cost ten clock cycles in all. The real time consumption can be decreased by parallelizing the actions mentioned above.

In front of every absolute difference (AD) calculator, there is a multiplexer which separates the 266 bytes left image data into two groups with the offset of 32 × 5 = 160 bytes. As an example, if the In1A is *P_L_*(0,0) which is the first pixel of the 5 × 5 window centered at *P_L_*(2,2), then the In1B should be assigned to *P_L_*(0,32) which is the first pixel of the 5 × 5 window centered at *P_L_*(2,34). Under the control of the SW signal, we use 32 SAD processing modules to determine the 64 SAD values separated in six clock cycles.

The basic comparison element (BCE) compares two input SAD values and produces a comparison bit served as a select signal for a multiplexer. The multiplexer outputs the minimum SAD involved in the comparison in the next stage. 63 BCEs constitute the disparity segregator as shown in Figure 7.

## 5. Design Principle of the Stereo Matching Controller

The controller is the commander of the stereo matching processing machine with three main functions listed below:

(a) Line buffer management.
(b) Disparity writes back.
(c) Stereo matching process control.

### 5.1. Line Buffer Management

There are two DPRAMs placing in the FPGA. Each has 1,024 × 16 = 16,384 bytes of memory space, acting as line buffers for stereo pairs. The controller initializes and updates the line buffers using the data read from the image frame buffers in the off-chip SSRAM connected by the Avalon-MM read master interface. The line buffers always store 16 lines of image data under the condition that the maximum amount of pixels per line is lower than 1,024. The direct mapping method is used to locate the pixel address in the line buffer. The mapping formula is shown as follows:

(2) J=(Ymod16)∗LinePixel+X

where *J* is the pixel address in the line buffer, *X* and *Y* are the pixel coordinates of the image, Linepixel is the number of horizontal pixels.

**Figure 8.**
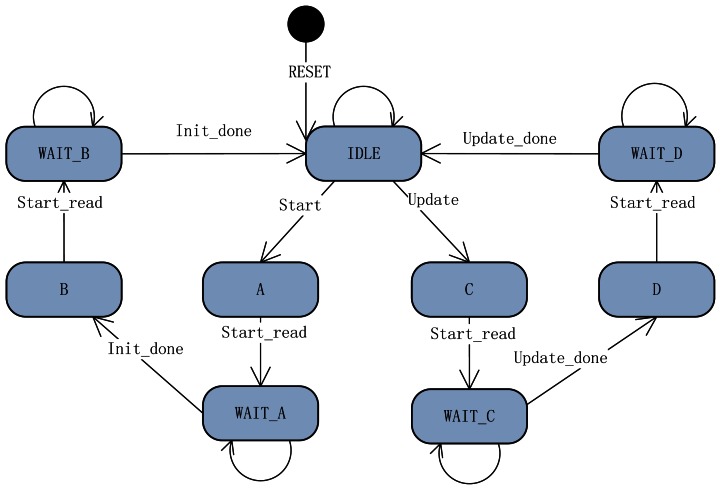
Finite State Machine of the Line Buffer Management.

The line buffer management process will be explained through the Finite State Machine (FSM) in Figure 8 and Table 1. There are 9 states that build the finite state machine for line buffer management in the SMC. The FSM of the line buffer management is activated by two signals, the start signal of the system and the update signal of the SMC. With the set action of the start signal, the FSM reads 16 lines of pixels at the beginning of the stereo images separately for initializing the two line buffers fully. After initialization of the line buffers, the FSM comes into idle state. The update signal activates the FSM into the updating buffer state. The FSM reads one word (4 byte pixel data) from every image in the SSRAM and replaces the oldest 4 pixels in line buffers, and then becomes idle again.

### 5.2. Disparity Write Back

When the system finishes the computation of the disparity of a pixel, a direct memory access controller is invoked to write it to the disparity table. For fear of conflicting with the reading action on the SSRAM caused by the line buffer update event, the dense disparity table is located in the off-chip DDRII SDRAM. The write back address is generated by the Equation (3):

(3) Addr=D_ADDR+2∗(Y∗LinePixel+X)

where *Addr* is the write back address, D_ADDR is the base address in the DDRII of the disparity table input by the Nios II processor, *X* and *Y* are the pixel coordinates, Linepixel is the number of horizontal pixels, the multiplication by 2 indicates every disparity value is occupying two bytes of memory.

### 5.3. Stereo Matching Process Control

**Figure 9.**
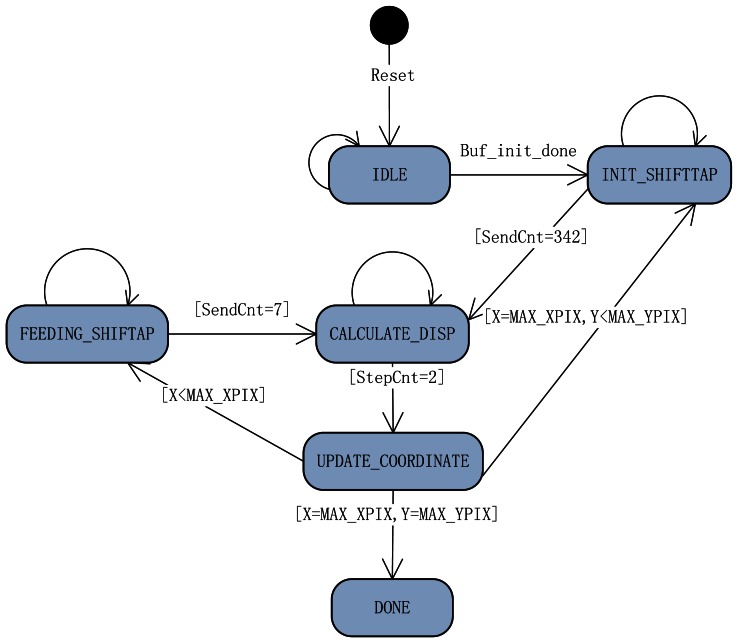
Finite State Machine of the Stereo Matching Process Control.

This is the most significant function among other blocks in the whole system. The process control is performed by a FSM involving only six states. Each state has different tasks. In the following list, the details of the tasks performed in each of the states are described. Figure 9 is the FSM diagram of stereo matching process control.

(a) After system reset, the FSM enters into the IDLE state automatically. In this state, all variables are initialized.
(b) After two line buffers are initialized with 16 lines of pixels, the FSM transfers into the INIT_SHIFTTAP state. Twenty five data bytes of the right line buffer and 340 data bytes of the left line buffer are read out and sent into the shift-taps concurrently in this state, and then the state comes into the CALCULATE_DISP state.
(c) In the CALCULATE_DISP state, the stereo machine spends two clock cycles setting switch signal and sending an activation signal to awake the latching data module. The latching data module is in charge of latching the 64 SAD values and finds the disparity from them, then writing it to the DDRII SDRAM. The module is executed concurrently with this FSM, therefore the time consumed by waiting for computing the disparity is decreased from 10 clocks to 2 clocks.
(d) In the UPDATE_COORDINATE state, the variables *X* and *Y*, the currently processed pixels' coordinate, are updated. There are three conditions for state transition in this state: (1) if *X* is smaller than the number of horizontal pixels, the next state is FEEDING_SHIFTTAP; (2) if *X* is equal to the number of horizontal pixels and Y is smaller than the number of vertical pixels, the next state is INIT_SHIFTTAP; (3) if *X* is equal to the number of horizontal pixels and *Y* equal to the number of vertical pixels, the next state is DONE.
(e) The task of the FEEDING_SHIFTTAP state is reading 5 bytes of new pixel data from each line buffer and sending them into the shift-taps, and then changing to the CALCULATE_DISP state.
(f) The DONE state is indicating that the whole dense disparity map is generated.

## 6. Results and Discussion

**Figure 10.**
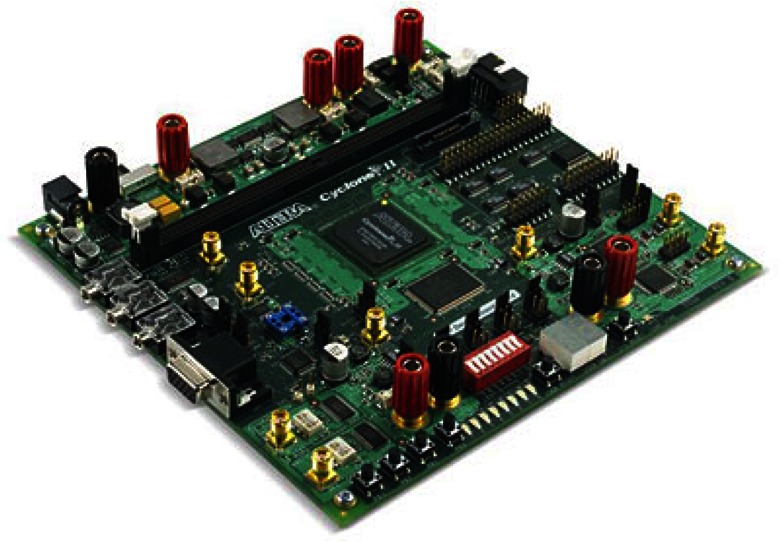
The DSP Development Kit, Cyclone II Edition Boar.

The stereo matching circuit has been realized by using an Altera Cyclone II EP2C70F672C6N device which is assembled on the Altera DSP Development kit Cyclone II Edition Board as shown in Figure 10. It is clocked with an external crystal of 100 MHz frequency.

**Figure 11.**
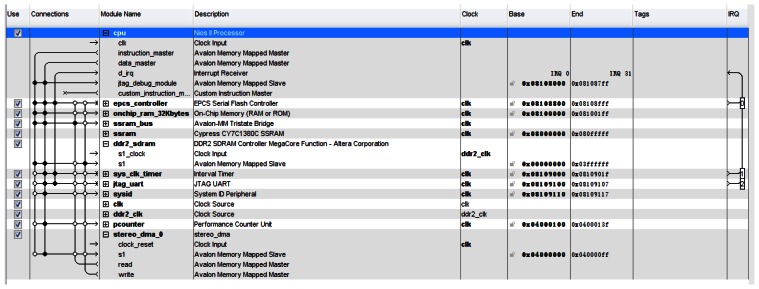
Screenshot of the Nios II System.

Figure 11 is a screenshot of the Nios II system designed in the SOPC Builder of Quartus II software. In Figure 11, the item named “stereo_dma_0” shows as an IP package including the module DCU and SMC mentioned in part 4 and part 5. The item “pcounter” is a performance counter unit used to measure the consumption time for processing a disparity map. The Performance counter, the only mechanism available with the Nios II development kits, provides measurements with little intrusion [10].

Table 2 shows the resources required from the FPGA device in order to implement the designs presented in this paper. The report is produced by the Quartus II v9.0 edition.

**Figure 12.**
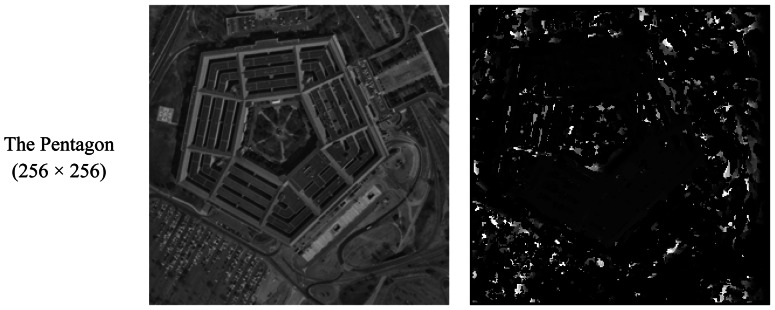

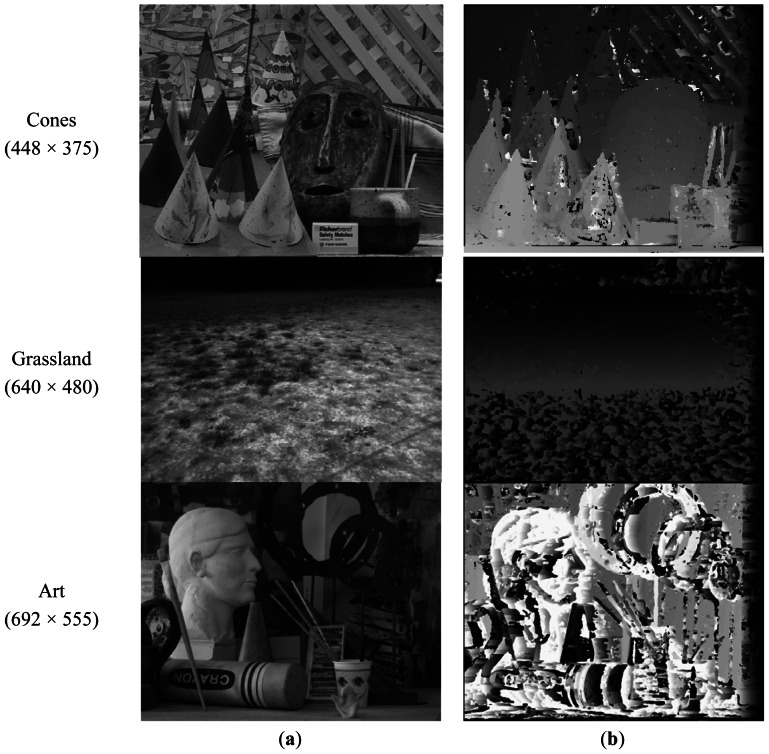
Examples: (**a**) the original right image; (**b**) the disparity map computed.

Several tests have been performed. The examples of disparity maps obtained by the machine are shown in Figure 12. Disparities are encoded using a scale factor of 4 for gray levels 0 to 254. Therefore, the encoded disparity range is 0 to 63 pixels.

The proposed system enables a suitable method for use in real-time demanding applications. The Table 3 gives the processing speed for the stereo pair images with different resolutions.

In Table 4, the proposed system is compared to the existing approaches in terms of speed.

In [11], the author implements a 3 × 3 SAD stereo matching software by using Intel's OpenCV library. The test platform is an Intel Pentium 4 with 3GHz clock frequency and 1GB memory. The processing time for one disparity map is 391 ms. This is 34 times slower than the proposed system. Kalomiros [12], whose SAD system can achieve 162 fps with assistance of a host computer, compares the SAD algorithm with dynamic programming algorithm. The system in paper [13] is designed for the detection of moving objects via using a stereo vision method, and it can get 30 images (640 × 480 pixels) in one second, but its maximum disparity of 27 is not seen as sufficient for many stereo vision applications.

## 7. Conclusions

An efficient hardware implementation of a real-time stereo matching processing machine is proposed by using an FPGA for the calculation of disparity maps. It takes full advantage of the convenience of IP reuse based on a SoPC architecture. It performs enough for stereo vision applications by means of a large disparity range. The frame rate could enable real time performance under the resolution of 640 × 480. The memory size is suitable for much higher resolution stereo pair images. The results for our system are very promising and may improve in the future. The system has been implemented on static image input from C code in the Nios II processor. We plan to incorporate live stereo video streams and combine the algorithm with pre- and post-processing stages to make it more suitable for the operation in robot auto- navigation and visual servo applications.

Furthermore, the main weakness of the SAD algorithm becomes evident in the FPGA implementation. As shown in Figure 12, SAD disparity maps in most cases have noise spots, especially in image areas of low light intensity. So we also intend to apply other robust stereo matching algorithms in our system, such as CENS, dynamic programming and Belief-Propagation, in our future works.

## Figures and Tables

**Table 1.** List of States.

**No.**	**States**	**Remark**	**Condition**
1	IDLE	Idle	Reset = 1
2	A	Initialization line buffer of right image with 16 lines of pixels	Start = 1, State = idle
3	B	Initialization line buffer of left image with 16 lines of pixels	Init_done = 1, State = WAIT_A
4	C	Update line buffer of right image 1 pixel data	Update = 1, State = idle
5	D	Update line buffer of left image 1 pixel data	Update_done =1, State = WAIT_C
6	WAIT_A	Waiting for Initialization line buffer of right image	Start_read = 1, State = A
7	WAIT_B	Waiting for Initialization line buffer of left image	Start_read = 1, State = B
8	WAIT_C	Waiting for updating line buffer of right image	Start_read = 1, State = C
9	WAIT_D	Waiting for updating line buffer of left image	Start_read = 1, State = D

**Table 2.** Resources needed for the Implementation of the Stereo Vision Machine.

Device
Altera EP2C70F672C6N (Cyclone II device family)
Resource
Total logic elements 49713/68416 (73%)
Total combinational functions 46131/68416 (67%)
Dedicated logic registers 15534/68416 (23%)
Total registers 15679
Total pins 115/422 (27%)
Total memory bits 389248/1152000 (34%)
Embedded Multiplier 9-bit elements 9/300 (3%)
Total PLLs 2/4 (50%)

**Table 3.** Processing Speed.

**Image**	**Image Size**	**Max Disparity**	*Total Clocks	*Fps	**Work Freq**
Pentagon	256 × 256	64	868766	103	90 MHz
Cones	448 × 375	64	2140161	42	90 MHz
Grassland	640 × 480	64	3848379	23	90 MHz
Art	692 × 555	64	4794308	18	90 MHz

* The total clocks are measured by the performance counter in the Nios II system.

* *Fps* = (90 × 10^6^)/(*Total clocks*).

**Table 4.** Comparison of Stereo Matching Implementations.

**Authors**	**Frame Rate**	**Image Size**	**Max. Disp**	**Algorithm**	**Window Size**	**Platform**
Motten *et al.* [8]	56 fps	800 × 600	80	SAD	7 × 7	1FPGA
Proposed impl.	23 fps	640 × 480	64	SAD	5 × 5	1 FPGA
Software impl. [11]	2.55 fps	320 × 240	100	SAD	3 × 3	PC
Kalomiros *et al.* [12]	162 fps	640 × 480	64	SAD	3 × 3	1FPGA + PC
Niitsuma *et al.* [13]	30 fps	640 × 480	27	SAD	7 × 7	1 FPGA
Miyajima *et al.* [14]	18.9 fps	640 × 480	80	SAD	7 × 7	1FPGA + PC
